# Multidrug-Resistant *Enterococcus faecalis* from Yak Feces: Isolation, Genomic Characterization and Functional Insights

**DOI:** 10.3390/vetsci12111077

**Published:** 2025-11-12

**Authors:** Jiayan Huang, Zixuan Li, Zhanchun Bai, Sizhu Suolang

**Affiliations:** College of Animal Science, Xizang Agricultural and Animal Husbandry University, Linzhi 860000, China; 202200201099@stu.xza.edu.cn (J.H.); 202200201096@stu.xza.edu.cn (Z.L.); baizhanchun@xza.edu.cn (Z.B.)

**Keywords:** yak, multidrug-resistant *Enterococcus faecalis*, multi-omics

## Abstract

*Enterococcus faecalis* (*E. faecalis*) is a significant zoonotic pathogen, primarily causing opportunistic infections in humans while often existing as a commensal in animal reservoirs, facilitating its dissemination. Current understanding of the resistance profiles, virulence mechanisms, and host–pathogen interactions of *E. faecalis* from ruminants, particularly unique species such as the plateau yak, remains limited. This knowledge gap hinders the accurate assessment of their transmission risk and the development of effective control strategies. This study presents a comprehensive analysis of a multidrug-resistant *E. faecalis* isolate from yak feces, integrating whole-genome sequencing (WGS), an animal challenge model, and transcriptomic profiling. Antimicrobial susceptibility testing revealed resistance to β-lactams, aminoglycosides, glycopeptides, tetracyclines, and fluoroquinolones. WGS identified numerous resistance genes (e.g., *parC*, *gyrA*, *rpoB*) and virulence-associated genes (e.g., *prgB/asc10*, *cpsA/uppS*). Phylogenetic analysis indicated a close relationship with a human urinary tract isolate (ASM3679337v1). Mouse challenge experiments demonstrated that this strain induced significant intestinal histopathological damage. A subsequent transcriptomic analysis of infected tissues identified the differential activation of key signaling pathways, including NF-κB and MAPK. Our findings provide crucial insights into the resistance and pathogenic mechanisms of ruminant-derived *E. faecalis* and establish an experimental foundation for optimizing clinical antimicrobial therapy against such strains.

## Simple Summary

*Enterococcus faecalis*, a “pan-host” commensal, is prevalent in the gut of humans and animals. Multidrug-resistant (MDR) strains from livestock, such as pigs and cattle, serve as a key reservoir for and transmission route of antibiotic resistance genes, posing a severe global threat to public health. Herein, we characterized an MDR *E. faecalis* isolate from yak feces using whole-genome sequencing, animal challenges, and transcriptomics to decode its resistome, virulome, and host transcriptional responses during infection. We established a “genotype–phenotype–host response” model for a ruminant-derived strain, offering fundamental insights into its resistance evolution and pathogenesis, which is vital for developing targeted antimicrobial strategies and reducing zoonotic transmission risks.

## 1. Introduction

The yak, an iconic endemic livestock species of the Qinghai–Tibet Plateau, serves not only as a core economic resource for local herders, but also plays an irreplaceable role in maintaining the stability of the alpine grassland ecosystem [1]. The fecal microbiota, acting as an “externalized carrier” of the gut microbial community, can reflect real-time changes in intestinal health, microbial homeostasis, and host physiological adaptation. Therefore, it is considered an ideal “non-invasive window” for studying yak gut microecological function.

*Enterococcus faecalis* (*E. faecalis*) is an important commensal bacterium in the gastrointestinal tracts of humans and various animals, working alongside other microbial communities to maintain gut microecological homeostasis and barrier function [2,3]. However, when the host intestinal barrier is compromised or long-term use of broad-spectrum antibiotics increases selective pressure, *E. faecalis* can translocate across the intestinal epithelium, invade distant organs such as the blood, liver, and heart, and subsequently cause systemic infections [4,5,6,7]. In clinical settings, *E. faecalis* has become one of the major opportunistic pathogens responsible for nosocomial infections, leading to various severe diseases including endocarditis [8], urinary tract infections [9], periapical periodontitis [10], and sepsis [11]. The prognosis of these infections is often influenced by their antibiotic resistance. Concurrently, the detection rate of multidrug-resistant (MDR) *E. faecalis* is generally high (≥88%) in intensively farmed animals such as pigs, cattle, and chickens, with most strains carrying mobile resistance genes. These resistant strains can be transmitted to humans through the environment, food chain, and other pathways, ultimately achieving cross-host transmission and significantly increasing the public health risk associated with controlling resistant bacteria [12,13]. The resistance of *E. faecalis* to commonly used clinical antibiotics exhibits a dual characteristic of being both “intrinsic and acquired.” This broad-spectrum resistance not only increases the difficulty of treating human clinical infections, but also limits clinical treatment options for animal-origin *E. faecalis* infections, easily leading to therapeutic failure. Furthermore, virulence factors carried by *E. faecalis* (such as the surface protein Esp [14], which promotes biofilm formation and intestinal colonization) can significantly enhance its adhesion, invasion, and immune evasion capabilities, further exacerbating its pathogenicity. The synergistic effect of this resistance and virulence poses a key challenge currently faced by both veterinary medicine and public health.

Research on *E. faecalis* has primarily focused on human and common livestock sources. The biological characteristics, resistance profiles, virulence, and molecular mechanisms of yak-derived *E. faecalis* remain unclear. To better understand the molecular biology of *E. faecalis*, the current mainstream approach involves the whole-genome sequencing analysis of bacterial strains. Whole-genome sequencing enables the functional annotation of bacteria through the analysis of virulence and antibiotic resistance genes, among other aspects. To further investigate pathogenicity-related indicators of this *E. faecalis* strain, subsequent animal challenge experiments were conducted using the strain, combined with transcriptomic analysis. Host tissue transcriptomics serves as a critical link between the bacterial strain and the host. By screening key differentially expressed genes and analyzing enriched related pathways, it can reveal the molecular mechanisms of bacterial invasion and colonization in target organs, providing data support for further research into the pathogenic mechanisms of *E. faecalis*.

## 2. Materials and Methods

### 2.1. Sample Source

In May 2023, a fresh fecal sample was collected from a 1–3-month-old neonatal yak calf with diarrhea, which was raised by a local household in Nyingchi City, Tibet Autonomous Region of China. All samples were aseptically collected using sterile swabs: each swab was gently inserted approximately 2 cm into the rectal cavity of a diarrheic yak to obtain feces, immediately placed into a 50 mL sterile centrifuge tube containing 10 mL of sterile phosphate-buffered saline, and transported on ice to the laboratory within 1.5 h of collection. These aseptically collected fecal samples were used for subsequent pathogen isolation.

### 2.2. Bacterial Isolation and Identification

Fecal samples were directly streaked onto blood agar plates and incubated at 37 °C for 24 h. Suspected single colonies were selected and identified as *Enterococcus* species using the microbiological and biochemical standard methods, including catalase testing, growth in 6.5% NaCl, and growth on bile–esculin agar, as described by Soltani et al. [15]. The confirmed *Enterococcus* isolates were then subcultured onto fresh blood agar plates and incubated at 37 °C for another 24 h. The bacterial lawns were harvested and suspended in sterile physiological saline. The resulting bacterial suspensions were serially diluted 10-fold. Subsequently, 100 μL of each dilution was spread onto new blood agar plates. After incubation under the same conditions, the colonies were counted for subsequent quantitative analysis. Additionally, all confirmed isolates were inoculated into LB broth containing 20% glycerol and stored at −80 °C for long-term preservation.

### 2.3. PCR Amplification and Identification

Bacterial DNA was extracted using the boiling method [16]. *E. faecalis* was confirmed and identified by PCR using the primers listed in Table 1. The primers were synthesized by Sangon Biotech (Shanghai, China) Co., Ltd. The 25 μL PCR reaction mixture consisted of 1 μL (10 μM) of each forward and reverse primer, 12.5 μL of 2X TaKaRa Taq™ Version 2.0 plus dye, 2 μL of DNA template (~50 ng/μL), and nuclease-free water added to a final volume of 25 μL. This setup resulted in a final concentration of 0.4 μM for each primer.

The amplification conditions were as follows: initial denaturation at 95 °C for 3 min, followed by 30 cycles of denaturation at 95 °C for 30 s, annealing at 52 °C (*E. faecalis*) and 53 °C (*Enterococcus genus*) for 30 s, and extension at 72 °C for 1 min, with a final extension at 72 °C for 7 min. The PCR amplification products were separated via electrophoresis on a 2% (*w*/*v*) agarose gel prepared in 1× TAE buffer. Electrophoresis was carried out at a constant voltage of 120 V for approximately 35 min. The gel was stained with ethidium bromide and visualized under UV light using a Gel Documentation System (Model: GelDoc XR+, Bio-Rad, Hercules, CA, USA). A 2000 bp DNA ladder (TaKaRa, Otsu, Shiga, Japan) was used as a molecular weight standard.

### 2.4. Whole-Genome Sequencing and Bioinformatic Analysis

#### 2.4.1. Genomic DNA Extraction, Quality Control, and Library Construction

The genomic DNA was extracted using the Cetyltrimethyl Ammonium Bromide (CTAB) method, and then the DNA concentration, quality, and integrity were determined using a Qubit Fluorometer (Invitrogen, Carlsbad, CA, USA) and a NanoDrop Spectrophotometer (Thermo Scientific, Waltham, MA, USA). Sequencing libraries were generated using the TruSeq DNA Sample Preparation Kit (Illumina, San Diego, CA, USA) and the Template Prep Kit (Pacific Biosciences, Menlo Park, CA, USA). The genome sequencing was performed by Personal Biotechnology Co., Ltd. (Shanghai, China). Sequencing libraries were constructed and subjected to both second-generation sequencing using the Illumina NovaSeq platform for high-throughput short-read data and third-generation single-molecule sequencing using the Oxford Nanopore platform (model: PromethION) for long-read data.

#### 2.4.2. Genome Assembly

Data assembly proceeded after adapter contamination removal and data filtering using AdapterRemoval [18] and SOAPec [19]. The filtered reads were assembled by SPAdes [20] and A5-miseq [21] to construct scaffolds and contigs. Flye [22] and Unicycler [23] software were used to assemble the data obtained through Nanopore platform sequencing. Subsequently, all assembled results were integrated to generate a complete sequence. Finally, the genome sequence was acquired after rectification using pilon software [24].

#### 2.4.3. Gene Prediction and Annotation

Genome function element prediction included the prediction of coding genes, non-coding RNA, and clustered regularly interspaced short palindromic repeats. Gene prediction was performed with GeneMarkS v4.32 [25]. tRNAscan-SE [26], Barrnap (version 0.9), and Rfam [27] were used to find tRNA, rRNA, and other ncRNA, respectively. CRISPRs were identified using a CRISPR recognition tool [28]. Repeat sequences were analyzed using RepeatModeler 2.0.7 software [29]. The RepBase database was used to predict sequences similar to known repeat sequences. Subsequently, the VFDB (Virulence Factors of Pathogenic Bacteria) database [30] and CARD (Comprehensive Antibiotic Resistance Database) [31] were used to retrieve the pathogenicity genes and antibiotic resistance genes, respectively.

### 2.5. Phylogenetic Analysis

All single-copy gene families were individually aligned using MUSCLE (http://www.drive5.com/muscle/; accessed on 3 December 2024). The resulting alignments were then concatenated to form a super alignment matrix. Finally, a maximum likelihood (ML) phylogenetic tree was constructed from the sequence alignment using RAxML (http://sco.h-its.org/exelixis/web/software/raxml/index.html; accessed on 6 November 2025).

### 2.6. Transmission Electron Microscopy (TEM) Observation

The bacterial stock solution described in Section 2.4 was revived by streaking onto blood agar plates. Single colonies were inoculated into 5 mL of LB broth and cultured at 37 °C for 24 h. The bacterial culture was then centrifuged at 12,000 r/min for 3 min, and the pellet was gently washed three times with PBS. The resulting bacterial cells were sent to the Experimental Technology Center at West China, Sichuan University. Subsequent sample processing and TEM (Hitachi, Tokyo, Japan, model HT7,800) observations were performed using the center’s facilities (including the Biosample Preprocessing Platform and Electron Microscopy Laboratory).

### 2.7. Antimicrobial Susceptibility Testing

The disk diffusion method was employed to assess the susceptibility of the *E. faecalis* isolate to a panel of antibiotics. The selection of antimicrobial agents, including penicillin (10 U), gentamicin (120 μg), kanamycin (30 μg), cefazolin (30 μg), ceftazidime (30 μg), cefradine (30 μg), vancomycin (30 μg), erythromycin (15 μg), tetracycline (30 μg), ofloxacin (30 μg), clindamycin (30 μg), and furazolidone (30 μg), was based on their clinical relevance for enterococcal infections and local resistance epidemiology data [32,33]. Testing was performed following the guidelines established by the Clinical and Laboratory Standards Institute [34].

### 2.8. Mouse Challenge Experiment and Histopathological Observation

Ten six-week-old specific pathogen-free (SPF) Kunming mice (half male and half female, average body weight ~32 g) were purchased from Sichuan Dashuo Experimental Animal Co., Ltd. and acclimatized for 10 days under isolated conditions. The frozen bacterial stock was thawed and subsequently cultured for 24 h with reference to the method described in Section 2.2. These mice were then orally administered 0.2 mL of a bacterial suspension at a concentration of 1 × 10^8^ CFU/mL, while the control group received an equal volume of physiological saline (*n* = 5 per group). Mouse health status and mortality were monitored and recorded every 12 h post-administration. Deceased mice were subjected to aseptic dissection with a focus on colon tissue collection, reflecting the specific tropism of *E. faecalis* for this site [35]. Colon tissues were fixed in 4% paraformaldehyde for 48–72 h, processed for hematoxylin and eosin (HE) staining by Wuhan Biofirm Biotechnology Co., Ltd., and examined under an optical microscope (Nikon, Japan, model ECLIPSE 80i). Additionally, colon tissues from aseptic dissection were used for bacterial re-isolation and identification.

### 2.9. Transcriptome Sequencing and Differential Gene Expression Analysis

At 72 h post-infection, colonic tissues were collected from both the *E. faecalis*-infected group and the uninfected control group, with three biological replicates per group (with each replicate representing an individual mouse). Immediately after dissection, the colonic tissues were briefly rinsed with pre-cooled sterile phosphate-buffered saline (PBS, pH 7.2) to remove residual intestinal contents, and then rapidly frozen in liquid nitrogen to prevent RNA degradation. Total RNA was subsequently extracted from the frozen colonic tissues, and transcriptome sequencing (RNA-seq) was carried out by Annoroad Gene Technology Co., Ltd. (Beijing, China) following the standard Illumina NovaSeq 6000 sequencing protocol (The frozen colon tissue samples were shipped on dry ice and delivered to the company within 24 h).

### 2.10. Data Analysis

#### 2.10.1. Differential Expression Analysis

For samples with biological replicates, differential expression analysis between comparison groups was performed using DESeq2 software. DESeq2 provides a statistical pipeline for identifying differentially expressed genes in digital gene expression data based on a model using the negative binomial distribution. The resulting *p*-values were adjusted using the Benjamini and Hochberg approach to control the false discovery rate [36]. Genes with an adjusted *p*-value ≤ 0.05 determined via DESeq2 were considered differentially expressed.

#### 2.10.2. Differential Gene Enrichment Analysis

Gene Ontology (GO) enrichment analysis and Kyoto Encyclopedia of Genes and Genomes (KEGG) pathway enrichment analysis of differentially expressed genes were conducted using the clusterProfiler software (model: version 4.0.0) [37]. GO terms and KEGG pathways with a *p* < 0.05 were defined as significantly enriched.

## 3. Results

### 3.1. Isolation and Identification of E. faecalis from Yak

**Figure 1:**
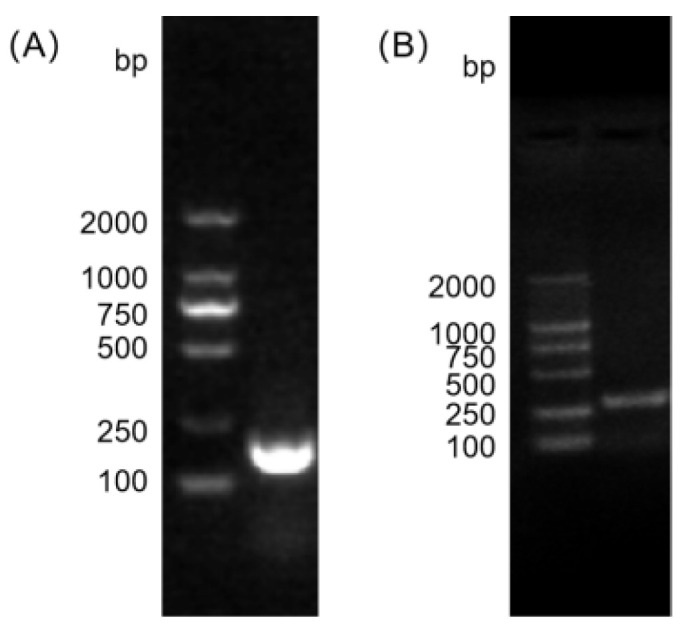
PCR amplification using *Enterococcus*-specific primers ((**A**), The original image is shown in Figure S1) and *E. faecalis*-specific primers ((**B**), The original image is shown in Figure S2).

A Gram-positive coccus was isolated from the yak fecal samples, which exhibited blue-violet coloration after Gram staining and morphological characteristics consistent with those of the *Enterococcus genus*. The biochemical identification results indicated that the isolate was catalase-negative and capable of growth in 6.5% NaCl and on bile–esculin agar. For molecular confirmation, PCR amplification was performed using genus-specific primers for *Enterococcus* and species-specific primers for *E. faecalis*. Electrophoresis on 2% agarose gel revealed distinct target bands at the expected molecular sizes for both primer sets (Figure 1), confirming that the isolated strain was indeed *E. faecalis*. The colonies from each dilution series were enumerated, and the results are shown in Table 2.

The bacterial concentration in the original solution was calculated to be 1.0 × 10^8^ CFU/mL.

### 3.2. Whole-Genome Sequencing Results

#### 3.2.1. General Genomic Features

The genome of the isolated *E. faecalis* strain was sequenced and found to be 2,885,327 bp in size, with a GC content of 37.49%, exhibiting a mean sequencing depth of 345×, a Q30 rate of 99.16%, and a contig N50 of 12,301 bp. It contained five prophage sequences and no plasmid sequences.

#### 3.2.2. Analysis of Antibiotic Resistance Genes

A total of nine categories of antibiotic resistance genes were identified. These included the fluoroquinolone resistance genes *parC*, *parE*, and *gyrA*, and the tetracycline resistance gene *EF3073*, among others (detailed results of the resistance gene analysis are presented in Table 3).

#### 3.2.3. Analysis of Virulence Genes

A total of seven categories of virulence system genes were detected. These included the pCF10 plasmid conjugation system gene *prgB/asc10*, pilus system genes *ebpB* and *ebpC*, and the Fsr quorum-sensing system genes *fsrA*, *fsrB*, *fsrC*, and *sprE*, among others (detailed results of the virulence gene analysis are presented in Table 4).

### 3.3. Phylogenetic Analysis

**Figure 2:**
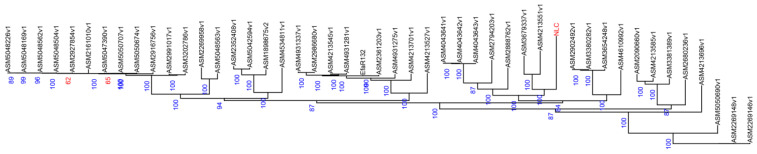
Phylogenetic tree based on genome assembly data. (The numbers represent support values, and the labels in red font (NLC) indicate the experimental strains).

As shown in Figure 2, the *E. faecalis* strain originating from yak clustered into a single, distinct monophyletic clade. This clade also contained isolates derived from human urine samples (e.g., strain ASM3679337v1, which showed the closest evolutionary distance to the NLC strain) and a human fecal sample (ASM4213551v1). The observed genetic relatedness suggests a potential shared lineage among these strains.

### 3.4. Transmission Electron Microscopy Observations

**Figure 3:**
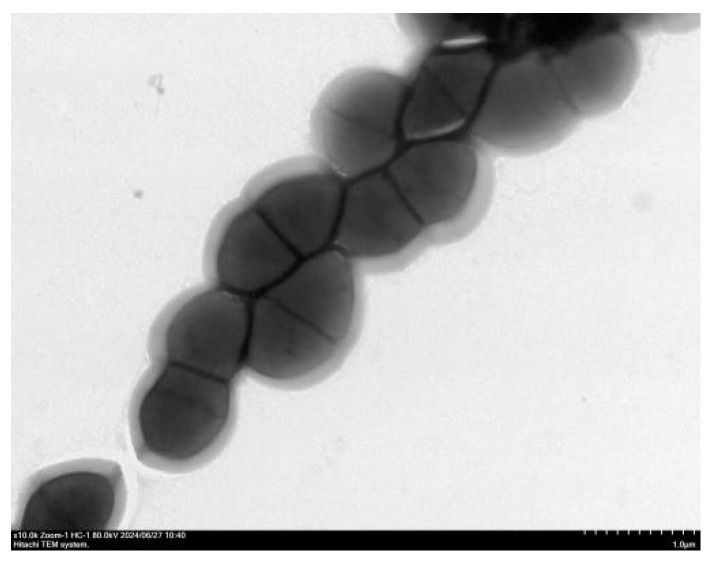
Transmission electron micrograph of *E. faecalis* (×10,000).

Under TEM, the bacterial cells appeared as spherical to ovoid cocci arranged in chains. The cell surface was smooth, with no flagella evident, but a putative capsule was observed (Figure 3).

### 3.5. Antimicrobial Susceptibility Testing Results

The isolated *E. faecalis* strain exhibited resistance to β-lactams (cefazolin, ceftazidime, cefradine), aminoglycosides (gentamicin, kanamycin), glycopeptides (vancomycin), tetracyclines (tetracycline), and fluoroquinolones (ofloxacin) (Table 5).

### 3.6. Mouse Challenge Experiment and Pathological Findings

**Figure 4:**
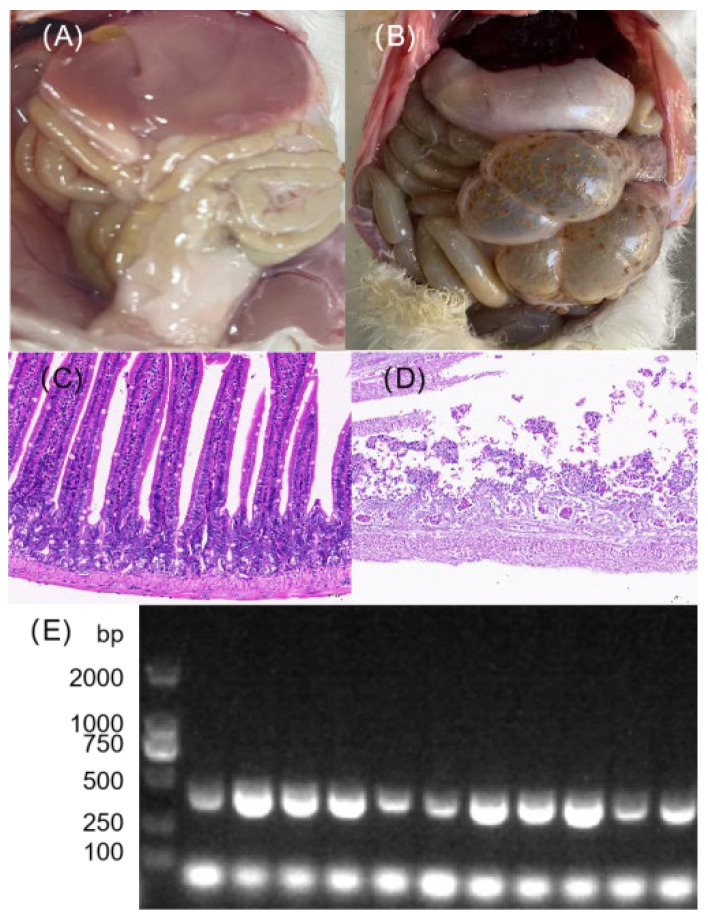
Pathological assessment of mice following challenge with the isolated bacterial strain. (**A**,**B**) Gross necropsy findings of the intestinal tract from (**A**) a control group mouse and (**B**) a mouse that died after bacterial challenge. (**C**,**D**) Representative HE-stained images of intestinal tissues from (**C**) control and (**D**) challenged mice (200× magnification). The infected group shows significant inflammatory cell infiltration and tissue damage. (**E**) PCR electrophoresis analysis of colonic tissues from the corresponding groups, confirming the presence of the challenged bacterium in the infected mice (The original image is shown in Figure S3).

Following challenge, the mice in the infected group exhibited varying degrees of morbidity within 1 to 5 days post-inoculation. Clinical signs included rapid breathing, reduced food intake, ruffled fur, huddling, decreased activity, increased ocular discharge, and diarrhea. No abnormalities were observed in the control group. Mortality began 24 h post-inoculation. The dissection results, as shown in Figure 4B, revealed intestinal manifestations including thinning of the intestinal wall, swelling, and translucency. Four of the five challenged mice died within the 9-day observation period.

Histopathological examination by HE staining revealed severe structural abnormalities in the colon tissue of the challenged group. The tissue exhibited extensive autolysis, with massive cellular degradation and only partial preservation of the intestinal architecture. In addition, extensive inflammatory cell infiltration was observed (Figure 4D). In contrast, the colon tissue of the control group maintained essentially normal overall structure. Colon tissues collected aseptically during necropsy from deceased mice were inoculated onto Columbia blood agar and incubated at 37 °C for 24 h. Bacterial identification was performed via PCR following the method described in Section 2.5. The 2% agarose gel electrophoresis results confirmed the presence of bands corresponding to *E. faecalis* in four colon tissue samples (Figure 4E).

### 3.7. Transcriptome Sequencing Results

After filtering, high-quality reads accounted for 97.36% of the raw sequencing reads. Of the total filtered bases, 95.29% possessed a quality score of Q30 or higher (indicating an error rate of less than 0.1%). Subsequent alignment to the reference genome showed high efficiency, with an average mapping rate of 96.49% in the control group (Control1: 97.23%; Control2: 97.04%; Control3: 95.21%) and 87.34% in the challenged group (NLC1: 70.90%; NLC2: 95.46%; NLC3: 95.67%).

#### 3.7.1. Differential Gene Statistics

**Figure 5:**
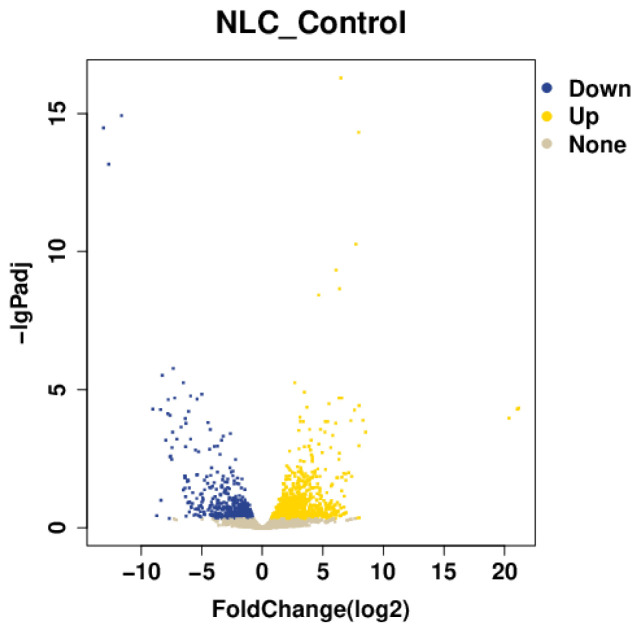
Volcano plot of differentially expressed genes.

A comparative analysis of colonic tissues between the challenged group and the control group identified a total of 2078 significantly differentially expressed genes (DEGs). Among these, 1346 genes were up-regulated and 732 were down-regulated (Figure 5).

#### 3.7.2. GO Enrichment Analysis

**Figure 6:**
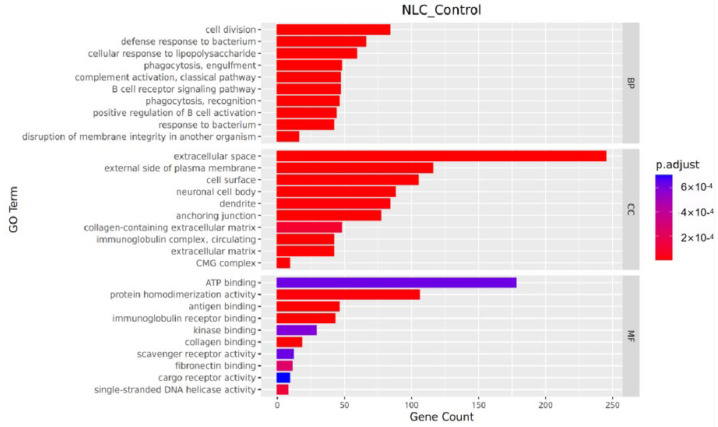
Bar plot of GO enrichment analysis.

In the molecular function category, significantly differentially expressed genes were primarily enriched for protein homodimerization activity, antigen binding, and immunoglobulin receptor binding. Within the cellular component category, significant enrichments were observed for the extracellular space, side of plasma membrane, and cell surface. For biological processes, the genes were mainly enriched in cell division, defense response to bacterium, and cellular response to lipopolysaccharide (Figure 6).

Significantly up-regulated genes were predominantly enriched in the functional categories of “Inflammatory and Immune Response” (e.g., *Nfkbia*), “Cellular Stress and Apoptosis” (e.g., *Fkbp5*), “Metabolic Reprogramming” (e.g., *Acot1*), and “Nervous System-related” (e.g., *Rtn4rl2*). In contrast, significantly down-regulated genes were enriched in “Protease Inhibition and Regulation” (e.g., *Serpina10*), “Metabolism” (e.g., *Scd2*), and “Fundamental Cellular Processes” (e.g., *Polq*) (Table 6).

#### 3.7.3. KEGG Pathway Analysis

**Figure 7:**
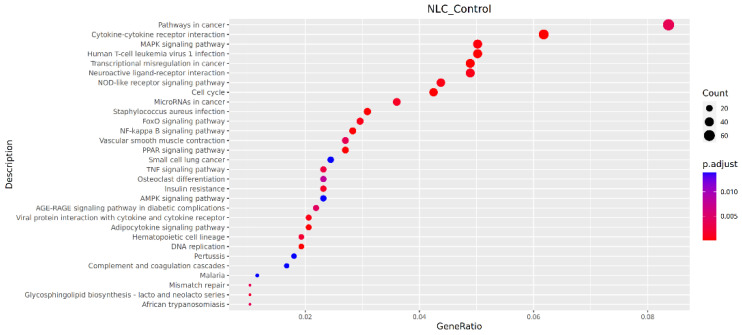
Bubble plot of KEGG pathway enrichment.

Pathways showing significant enrichment (*p* < 0.001) included Cytokine–cytokine receptor interaction, *Staphylococcus aureus* infection, DNA replication, Transcriptional misregulation in cancer, Cell cycle, PPAR signaling pathway, NF-kappa B signaling pathway, Adipocytokine signaling pathway, Human T-cell leukemia virus 1 infection, and MAPK signaling pathway (Figure 7).

## 4. Discussion

### 4.1. Antimicrobial Resistance and Virulence Risks Revealed by Genomic Characteristics

The prediction of antimicrobial resistance genes, specifically the *vanB* (carried by *EF0854*) [37,38] and *gyrA* mutations [39,40], identified core markers of clinically multidrug-resistant enterococci. The co-occurrence of *gyrA* and *parC* mutations exacerbates multidrug resistance [39], and this dual mutation can multiplicatively increase the risk of treatment failure. This suggests that the strain identified in this study, harboring both *gyrA* and *parC* mutations, may possess enhanced resistance capabilities. Consequently, its susceptibility profile requires priority consideration in clinical management to avoid therapeutic failure resulting from empiric antibiotic therapy.

The predicted virulence genes—*ebpB*, *ebpC*, *cpsA/uppS*, *fsrA*, *fsrB*, and *prgB/asc10*—belong to distinct pathogenic systems in *E. faecalis*. They act in concert to execute its core pathogenic mechanism: from adhesion and colonization, progressing to immune evasion, and then tissue invasion and destruction, further leading to the formation of resistant biofilms and ultimately enabling the efficient dissemination of virulence and resistance genes.

### 4.2. Validation of Concordance Between Phenotype and Genotype

Transmission electron microscopy revealed the presence of a capsule structure in this strain, which may enhance its ability to adhere to the intestinal mucosa. This morphological feature is complemented by the adhesion-associated virulence gene *EF0818* detected through whole-genome sequencing. The capsule and the adhesion gene likely act synergistically to enhance adhesion efficiency, providing a dual assurance—structural and molecular—for the strain to resist clearance and achieve stable survival within the intestinal tract. This synergy elucidates the mechanism underlying its colonization advantage.

Antimicrobial susceptibility testing demonstrated that the strain was resistant to five classes of commonly used clinical antibiotics: β-lactams, aminoglycosides, glycopeptides, tetracyclines, and fluoroquinolones. This resistance profile is concordant with the resistance-related genes and/or mutations identified through whole-genome sequencing.

### 4.3. Integrated Analysis of HE Staining Phenotypes and Transcriptomic Enrichment Results

The extensive inflammatory cell infiltration observed in HE staining finds clear mechanistic support at the transcriptomic level. GO enrichment analysis showed the significant up-regulation of the “Inflammatory and Immune Response” category, exemplified by the *Nfkbia* gene, while the KEGG pathway analysis revealed the significant activation of “Cytokine–cytokine receptor interaction” and the “NF-κB signaling pathway”. *Nfkbia*, a negative regulator of the NF-κB signaling pathway, binds to and sequesters NF-κB complexes (e.g., the p50/p65 dimer) in the cytoplasm upon its up-regulation, thereby preventing their nuclear translocation and subsequent activation of downstream target genes [41,42]. Notably, the enrichment of the “Staphylococcus aureus infection” pathway in KEGG shares the core mechanism of “NF-κB-mediated inflammatory regulation” with *E. faecalis* infection [43], a finding that underscores a shared pathogenic strategy among enterococcal pathogens in modulating host inflammation.

The severe histopathological phenotype observed in HE staining—characterized by widespread tissue autolysis and extensive cellular degradation—originates from the molecular foundations revealed by the transcriptome: the up-regulation of the “Cellular Stress and Apoptosis” GO category (e.g., *Fkbp5*) and aberrant activation of the “MAPK signaling pathway” and “Cell cycle pathway”. The up-regulation of *Fkbp5* promotes the release of inflammatory factors, disrupts intestinal barrier function, and induces apoptosis by activating the NF-κB signaling pathway [44], thereby exacerbating tissue damage and systemic inflammatory responses caused by *E. faecalis* infection. The MAPK signaling pathway serves as a critical hub transducing various stress signals into apoptotic responses. Concurrently, the aberrant enrichment of the cell cycle pathway suggests an impaired proliferative repair process in intestinal epithelial cells [45], ultimately manifesting as the residual intestinal architecture seen in HE staining. This dual mechanism—accelerated apoptosis coupled with diminished regenerative capacity—constitutes the core molecular basis of intestinal autolysis. Furthermore, transcriptomic enrichments—including the “Metabolic Reprogramming” GO term (e.g., up-regulated *Acot1*), the down-regulated “Protease Inhibition and Regulation” term (e.g., *Serpina10*), and the KEGG pathways “PPAR signaling pathway” and “Adipocytokine signaling pathway”—collectively explain the functional disruption following intestinal tissue damage.

This study, through an integrated pathology–transcriptomics analysis, revealed that the pathogenic mechanism of the yak-derived *E. faecalis* strain shares significant commonality with clinical human-derived strains: both primarily employ the core strategy of “activating the NF-κB/MAPK inflammatory pathways [46] and inducing cellular stress and apoptosis [47].” This finding is in agreement with the phylogenetic analysis result, wherein the yak-derived strain clustered closely with human-derived strains, thereby providing further support for the hypothesis of a potential risk for the cross-host transmission of *E. faecalis.* While the mouse model provides valuable insights into these fundamental mechanisms, we emphasize that the specific host-pathogen interactions within the natural yak host remain to be investigated and may involve additional, host-specific factors.

## 5. Conclusions

A strain of *E. faecalis* was successfully isolated and identified from yak feces. The isolate carries antibiotic resistance genes, including *parC* and *gyrA*, along with virulence genes such as *prgB/asc10* and *fsrA*. It exhibits resistance to β-lactams, aminoglycosides, glycopeptides, tetracyclines, and fluoroquinolones, indicating its potential pathogenicity. In a mouse challenge model, the strain induced intestinal inflammation. Transcriptomic analysis suggested that this pathogenesis may involve the up-regulation of *Nfkbia*, which binds to and sequesters NF-κB in the cytoplasm, preventing its nuclear translocation and activation of downstream target genes. Concurrently, the up-regulation of *FKBP5* may activate the NF-κB signaling pathway, promoting the release of inflammatory factors, disrupting intestinal barrier function, and inducing apoptosis. This study provides a scientific basis for the risk assessment and health management of *E. faecalis* in the yak intestinal tract.

## Figures and Tables

**Table 1:** Primers used in the PCRs carried out in this study.

Strain/Gene	Primer	Sequence (5′-3′)	Product Size (bp)	Reference
*Enterococcus genus*	tuf-F	TACTGACAAACCATTCATGATG	112	[17]
	tuf-R	AACTTCGTCACCAACGCGAAC		
*E. faecalis*	FL1	ACTTATGTGACTAACTTAACC	360	[17]
	FL2	TAATGGTGAATCTTGGTTTGG		

**Table 2:** Colony counts for quantitative analysis.

Log Dilution	Plate Count 1	Plate Count 2	Plate Count 3	$\bar{x}\pm s$
0	Countless	Countless	Countless	/
1	Countless	Countless	Countless	/
2	Countless	Countless	Countless	/
3	Countless	Countless	Countless	/
4	Countless	Countless	Countless	/
5	87	112	101	100 ± 25
6	15	23	31	23 ± 16
7	0	0	0	0

**Table 3:** Prediction of antibiotic resistance genes.

Resistance Category	Resistance Gene(s)	Resistance Mechanism
Fluoroquinolone resistance	*parC*, *parE*, *gyrA*	Gene mutations leading to structural alterations in the drug target protein
Macrolide–Lincosamide–Streptogramin (MLS)	*Lsa*, *emeA*	Ribosomal target site modification or active efflux of the drug
Rifampin resistance	*rpoB*, *rpoC*	Mutations in the drug target site leading to drug inactivation
Tetracycline resistance	*EF3073*	Steric hindrance blocking drug-ribosome interaction
Trimethoprim resistance	*dfrE*	Mutation of the target enzyme leading to drug inefficacy
“Target Bypass” mediating D-Cycloserine resistance	*alr*	Affects cell wall precursor synthesis

**Table 4:** Prediction of virulence genes.

Virulence System	Virulence Gene(s)	Core Pathogenic Mechanism
pCF10 Plasmid Conjugation System	*prgB/asc10*	Facilitates bacterial aggregation and host cell invasion
Fsr Quorum-Sensing System	*fsrA*, *fsrB*, *fsrC*, *sprE*	Regulates the expression of virulence factors (e.g., gelatinase encoded by *sprE*)
Capsular Polysaccharide Synthesis System	*cpsA/uppS*, *cpsB/cdsA*, *cpsC*, *cpsD*, *cpsE*, *cpsG*, *cpsH*, *cpsI*, *cpsK*	Encapsulates the bacterial surface to resist phagocytosis by host immune cells
Pilus System	*ebpB*, *ebpC*	Encodes pilus structures that mediate bacterial adhesion to host cells (e.g., endothelial cells)
Sugar-Binding Transcriptional Regulator	*bopD*	Injects effector proteins into host cells, triggering intracellular infection
Adhesin System	*EF0818*	Mediates bacterial aggregation, host cell adhesion, and biofilm formation
Adhesion System	*EF0485*	Encodes bacterial adhesion-associated proteins

**Table 5:** Disk diffusion antimicrobial susceptibility testing results.

Antimicrobial Agent	Disk Content (μg/Disk)	Interpretation Criteria (mm)			Inhibition Zone Diameter (mm)	Susceptibility
		R	l	S		
Penicillin	10 U	≤14	—	≥15	19	R
Gentamicin	120	≤6	7~9	≥10	NM	R
Kanamycin	30	≤13	14~17	≥18	NM	R
Cefazolin	30	≤14	15~17	≥18	13	R
Ceftazidime	30	≤14	15~17	≥18	NM	R
Cefradine	30	≤14	15~17	≥18	11	R
Vancomycin	30	≤14	15~16	≥17	NM	R
Erythromycin	15	≤13	14~22	≥23	NM	R
Tetracycline	30	≤14	15~18	≥19	12	R
Ofloxacin	5	≤12	13~15	≥16	10	R
Clindamycin	2	≤14	15~20	≥21	NM	R
Furazolidone	300	≤14	15~16	≥17	19	S

Abbreviations: R, resistant; I, intermediate; S, susceptible; NM, not measured (testing was performed following the guidelines established by the Clinical and Laboratory Standards Institute [34]).

**Table 6:** Functional categorization of significantly up-regulated and down-regulated genes.

Regulation	Gene Names	Functional Category
Up-regulated	*Cp*, *Mmp8*, *Itih3*, *Nfkbia*, *Serpina3n/k/c/f/m/h*, *Hdc*, *Ctsk*, *Dusp1*	Inflammatory and Immune Response
	*Gpx3*, *Chac1*, *Fkbp5*, *Asns*, *Akr1b8*, *Glul*	Cellular Stress and Apoptosis
	*Aldh1l2*, *Adhfe1*, *Pfkfb3*, *Hmgcs2*, *Acacb*, *Acot1*, *Glul*	Metabolic Reprogramming
	*Phox2a*, *Jph3*, *Apod*, *Pou3f3*, *Rtn4rl2*, *Kcns3*	Nervous System-related
Down-regulated	*Serpina1f*, *Serpina10*	Protease Inhibition and Regulation
	*Scd2*, *Suox*	Metabolism
	*Kif12*, *Polq*	Fundamental Cellular Processes
	*Gm16477*, *Rps4l*, *Gm5837*, *Gm46223*, *Rpsa-ps10*	Predicted Genes/Pseudogenes

## Data Availability

The data presented in this study are openly available in NCBI SRA at https://www.ncbi.nlm.nih.gov/sra/ (accessed on 11 July 2025), reference number SUB15393040/SAMN4953547/8/9.

## Supplementary Materials

The following supporting information can be downloaded at: https://www.mdpi.com/article/10.3390/vetsci12111077/s1, Figure S1: The original image of Figure 1A, Figure S2: The original image of Figure 1B, Figure S3: The original image of Figure 4.
