# Supplementary figures and images for: Multidrug-Resistant Enterococcus faecalis from Yak Feces: Isolation, Genomic Characterization and Functional Insights
Source: Vet Sci. 2025 Nov 12;12(11):1077. doi: 10.3390/vetsci12111077 (PMC12656877; doi:10.3390/vetsci12111077)

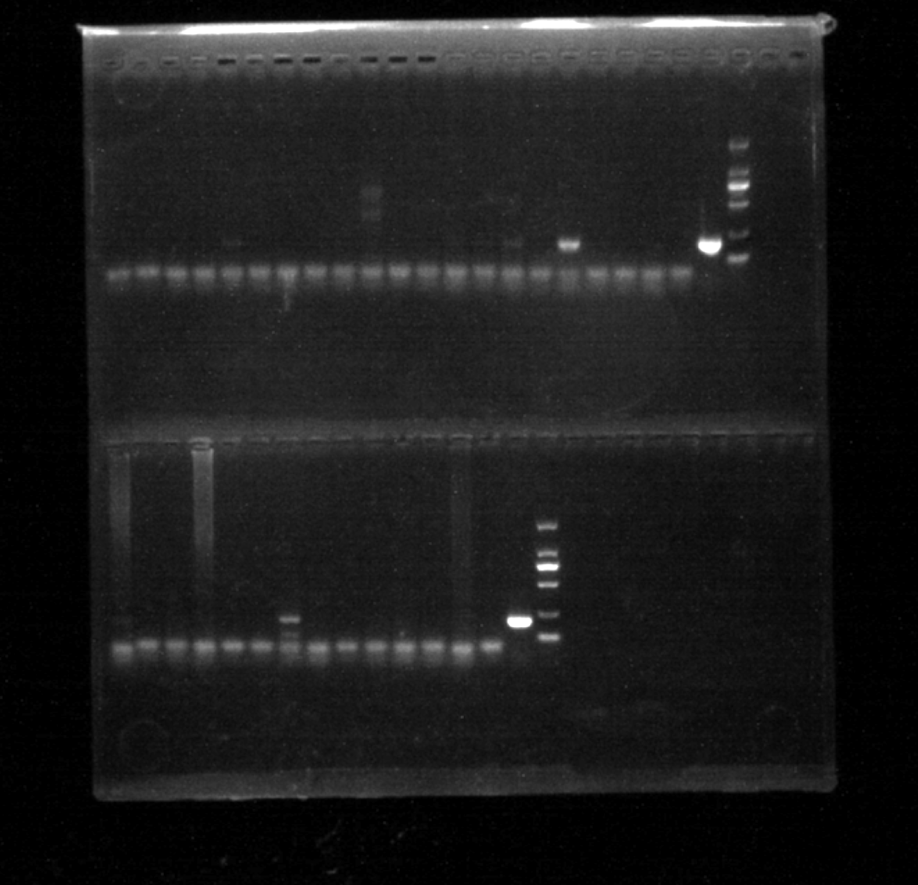

Supplement: Supplementary file 1 [file vetsci-12-01077-s001.zip › Figure S1.png]

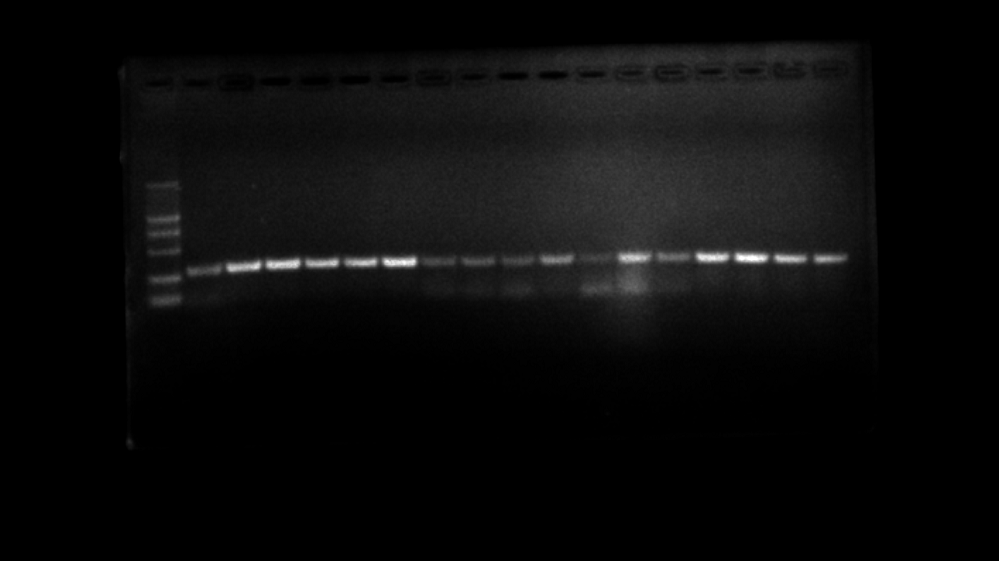

Supplement: Supplementary file 1 [file vetsci-12-01077-s001.zip › Figure S2.png]

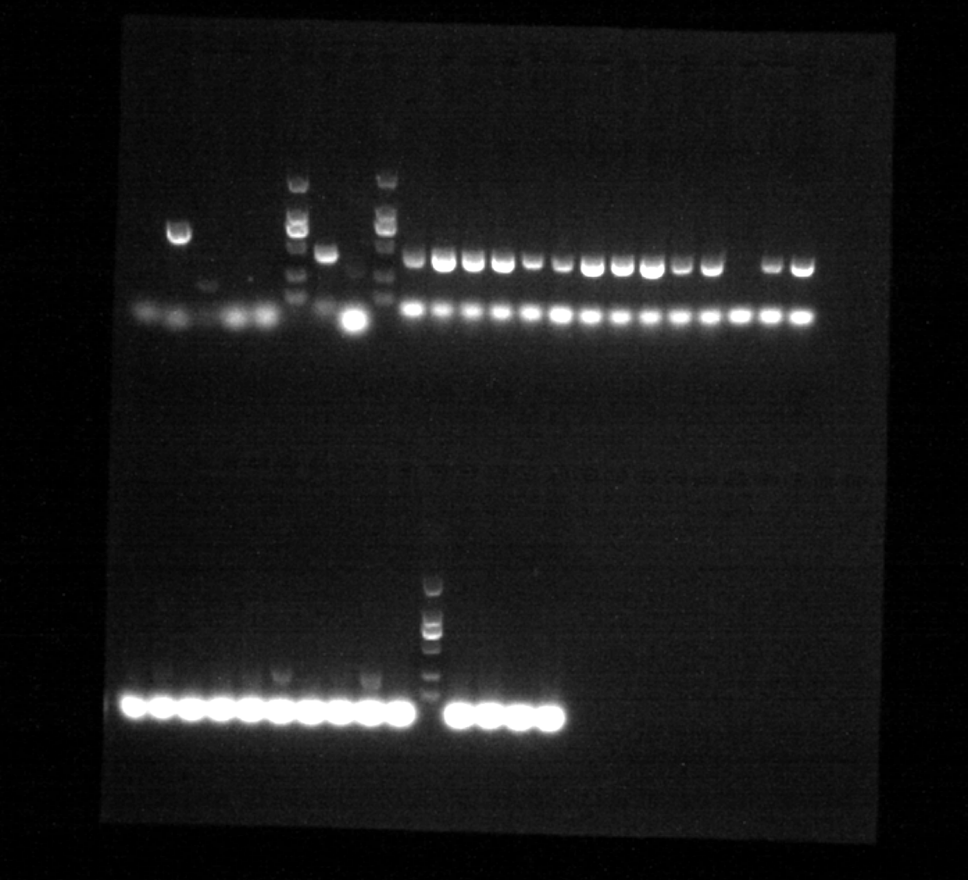

Supplement: Supplementary file 1 [file vetsci-12-01077-s001.zip › Figure S3.png]
